# Intragenic Variations in *BTLA* Gene Influence mRNA Expression of *BTLA* Gene in Chronic Lymphocytic Leukemia Patients and Confer Susceptibility to Chronic Lymphocytic Leukemia

**DOI:** 10.1007/s00005-016-0430-x

**Published:** 2016-12-08

**Authors:** Lidia Karabon, Anna Partyka, Monika Jasek, Ewa Lech-Maranda, Olga Grzybowska-Izydorczyk, Agnieszka Bojarska-Junak, Edyta Pawlak-Adamska, Anna Tomkiewicz, Tadeusz Robak, Jacek Rolinski, Irena Frydecka

**Affiliations:** 1 Department of Experimental Therapy, Hirszfeld Institute of Immunology and Experimental Therapy, Polish Academy of Sciences, Wroclaw, Poland; 2 Department and Clinic of Urology, Wroclaw Medical University, Wroclaw, Poland; 3 Department of Clinical Immunology, Hirszfeld Institute of Immunology and Experimental Therapy, Polish Academy of Sciences, Wroclaw, Poland; 4 Department of Hematology, Institute of Hematology and Transfusion Medicine, Warsaw, Poland; 5 Department of Hematology and Transfusion Medicine, Centre of Postgraduate Medical Education, Warsaw, Poland; 6 Department of Hematology, Medical University of Lodz, Lodz, Poland; 7 Department of Clinical Immunology and Immunotherapy, Medical University of Lublin, Lublin, Poland

**Keywords:** BTLA, Gene polymorphisms, mRNA expression, Chronic lymphocytic leukemia

## Abstract

The aim of this study was to determine the association between polymorphisms in gene encoding B- and T-lymphocyte attenuator (*BTLA*) and susceptibility to chronic lymphocytic leukemia (CLL) and their influence on mRNA expression of *BTLA* gene in T and B cells from CLL patients (pts.). The following *BTLA* single-nucleotide polymorphisms (SNPs): rs2705511, rs1982809, rs9288952, rs76844316, rs16859633, rs9288953, rs2705535, rs1844089, rs2705565, rs2633580 were genotyped with use of TaqMan probes in 321 CLL pts. and in 470 controls. The mRNA levels of human *BTLA* were determined in subpopulations of T and B cells from 37 CLL patients with use of Applied Biosystems assays. Three SNPs: rs1982809, rs2705511 and rs9288953 were associated with susceptibility to CLL. The frequency of rs1982809[G] allele and rs2705511[C] allele carriers was higher in patients compared to the controls (0.51 vs. 0.41, OR 1.51, 95% CI 1.14–2.02, *p* = 0.004 and 0.56 vs. 0.44, OR 1.62, 95% CI 1.22–2.16, *p* = 0.0009, respectively). Furthermore, rs9288953[TT] genotype was overrepresented in CLL pts. compared to the controls (0.22 vs. 0.14, OR 1.74, 95% CI 1.20–2.53, *p* = 0.004). The evaluation of the influence of *BTLA* SNPs on *BTLA* mRNA expression in CLL pts. showed that the presence of rs1982809[G] allele was associated with lower median (±SD) *BTLA* mRNA expression in T cells (expressed as 2-delta Ct) in CLL pts. as compared to [AA] homozygotes (0.009 ± 0.013 vs. 0.026 ± 0.012, *p* = 0.03). Our results indicate that rs1982809 *BTLA* gene polymorphism is associated with mRNA expression level and that variations in the *BTLA* gene might be considered as potentially low-penetrating CLL risk factor.

**Electronic supplementary material:**

The online version of this article (doi:10.1007/s00005-016-0430-x) contains supplementary material, which is available to authorized users.

## Introduction

A properly functioning innate and adaptive immunity provides effector cells such as lymphocytes and natural killer (NK) cells that are able to recognize and eliminate cells transformed into a cancer cells. The aberrant control of adaptive immunity can lead to insufficient tumor immune surveillance and to tumor development and progression. The activation of lymphocytes is regulated by co-signaling molecules which are also called “immune check-point” (Pardoll 2012), since the balance between their stimulatory and inhibitory signals determines regulation of immune response. To date, two major “immune check-point” molecules have been described: cytotoxic T lymphocytes antigen-4 (CTLA-4) and programmed cell death 1 (PD-1). Recently B- and T-lymphocyte attenuator (BTLA) was recognized as a potential “immune check-points” molecule.

BTLA is a type 1 membrane glycoprotein which is expressed on CD4^+^ and CD8^+^ T cells, B cells, NK T cells, NK cells, DCs and macrophages (Kobayashi et al. 2013; Watanabe et al. 2003). The over-expression of BTLA (as well as its ligand HVEM) was observed in cancers (Pasero and Olive 2013), especially in malignant T lymphoproliferative disorders (Karakatsanis et al. 2014). High expression of BTLA was also observed in B cell malignancies, in particular in chronic lymphocytic leukemia (CLL) (M’Hidi et al. 2009). Moreover, the simultaneous higher expression of HVEM and BTLA in CLL patients (pts.) was observed, what may suggest the triggering of an ineffective autocrine inhibitory loop. Additionally, the high expression of another co-inhibitory molecule PD-1 on CLL cells was reported (M’Hidi et al. 2009). It is postulated that CLL cells use the BTLA/HVEM and PD-1/PD-L1 pathways to inhibit T cell response and enhance their survival (Pasero and Olive 2013). Considering the important role of BTLA in CLL pathogenesis, we hypothesize that genetic variation in *BTLA* gene might be associated with CLL susceptibility and outcome.

Therefore, in the present study, we evaluated the association between ten single-nucleotide polymorphisms (SNPs) of *BTLA* gene chosen on the basis of literature survey, and in silico analysis as well as LD TagSNP selection with CLL risk and outcome. Additionally, we determined the *BTLA* mRNA expression in T and B cells from CLL pts. and analyzed the expression level in relation to genetic variations.

## Materials and Methods

### Patients

The study group comprised 321 (144 females and 177 males) pts. diagnosed with CLL originating from two main cohorts. The first cohort consisted of patients from Department of Haematology at the Medical University of Lodz, the Regional Oncology Center in Lodz (together 203 pts.), the other from the Department of Haematooncology and Bone Marrow Transplantation of Medical University of Lublin (111 pts.). The remaining seven patients were recruited from the Department of Haematology, Neoplastic Diseases and Bone Marrow Transplantation, Medical University, Wroclaw. Diagnosis of CLL was based on criteria from the International Workshop on Chronic Lymphocytic Leukaemia (Hallek et al. 2008). Patients’ characteristics are presented in Table 1.

**Table 1:** Baseline characteristics of 321 patients with CLL at the time of diagnosis

Characteristics	Patients	
	*N* (%)	Median (range)
Age (≤60 years/>60 years)	119 (37)/202 (63)	64 (38–85)
Gender (female/male)	144 (45)/177 (55)	
Hemoglobin (g/dL)		13.3 (4.4–17.3)
Platelet count (10^9^/L)		178 (3–745)
Rai stage (0–II/III–IV)	266 (83)/55 (17)	
β2M (normal/elevated/unknown)	48 (24)/156 (76)/117	2833 (950–12.790)
LDH (normal/elevated/unknown)	136 (65)/72 (35)/113	291 (94–1278)
CD38 [low (≤30%)/high (>30%)/unknown]	202 (73)/76 (27)/43	10.74 (0–100)
ZAP-70 [low (<20%)/high (≥20%)/unknown]	141 (75)/48 (25)/132	7.59 (0–74.94)
17 deletion (present/absent/unknown)	18 (9)/183 (91)/120	
*IGHV* mutation status (yes/no/unknown)^a^	97 (53)/85 (47)/21	
Treatment (yes/no/unknown)	180 (58)/133 (42)/8	
TFS		10 (0–123)
Death (yes/no/unknown)	38 (18)/178 (82)/105	
OS		
Death pts., time to death in months		49.5 (5–143)
Living pts. time to last examination in months		64 (1–259)

^a^Only in first cohort (203 pts.)

The details for the CD38 and ZAP-70 expression determination as well as 17p chromosome deletion and immunoglobulin heavy chain variable (IGHV) status (which was determined only in the first cohort) are described in detail in Supplementary material 1. All patients were followed up and time lag to the occurrence of at least one of the following events was recorded: doubling of peripheral lymphocyte count as compared to the initial value, progression to a higher Rai stage and the appearance of the indications for cytostatic treatment according to NCI-Sponsored Working Group recommendations. In 38 pts., the indications for treatment appeared and chlorambucil was given as front-line therapy to all pts. requiring treatment, and purine analogs-based protocols were used in the treatment of refractory/relapsed cases. Treatment-free survival (TFS) was calculated from the date of CLL diagnosis to the first treatment, death or the last follow-up if untreated. Overall survival (OS) was determined from the date of diagnosis until the last follow-up evaluation or death arising from any cause.

### Controls

The control population comprised 470 healthy subjects (206F/264M) originating from the same geographical area as the pts. recruited from the blood bank in Wroclaw or from employees of the Hirszfeld Institute of Immunology and Experimental Therapy. All participants gave written informed consent.

### Selection of SNPs

For this study, we have selected SNPs described previously in the literature: rs1844089, rs2705535, rs9288952, rs9288953, rs76844316, rs16859633 (Fu et al. 2010; Inuo et al. 2009; Lin et al. 2006; Oki et al. 2011) and additionally the tag dSNPs which covered entire *BTLA* gene together with 5000 bp upstream and 5000 bp downstream regions: rs1982809, rs2633580, rs2705511, rs2705565. The localization of all SNPs was previously described (Partyka et al. 2015). The tag SNPs selection was done with use of SNPinfo (Xu and Taylor 2009) and was based on the following criteria: SNP under linkage disequilibrium *r* ^2^ > 0.8 and available at the National Center for Biotechnology Information for Caucasian population of rare alleles at a frequency greater than 5%.

Both SNPinfo and FastSNP programs were used for SNPs function prediction (Xu et al. 2007; Yuan et al. 2006). According to in silico analysis, the following SNPs are located in the potential transcription factor binding sites: rs1844089, rs2633580, rs2705565, while rs9288952, rs76844316 and rs16859633 polymorphisms are missense mutations.

### Genotyping/Determination of Polymorphisms

DNA was isolated from venous blood according to the manual procedure for white blood cells using the QIAamp DNA Blood Mini Kit (Qiagen, Germany).

SNPs in the *BTLA* gene: rs1844089, rs2705535, rs9288952, rs9288953, rs1982809, rs2633580, rs2705511, rs76844316, rs16859633 were genotyped using TaqMan^®^SNP Genotyping Assays, respectively: C__26921149_20, C__16272852_ 10, C___1175845_10, C___1175838_10, C___1175848_20, C__16047575_10, C__16272823_10, C__34010634_10, provided by Applied Biosystems (Foster City, USA). Genotyping for rs2705565 was done using TIB MOLBIOL LightSNiP assay (no. 24901301).

### mRNA Study

The subpopulation of T and B cells was separated from frozen peripheral blood mononuclear cells from 37 CLL pts. The mRNA expression levels of human *BTLA* were determined using Applied Biosystems assays. The detailed procedure is presented in Supplementary material 2.

### Statistical Analysis

Hardy–Weinberg equilibrium (HWE) was evaluated independently for the patients and the controls by comparing the observed and expected frequencies of genotypes using *χ* ^2^ analysis. The *χ* ^2^ test was used to compare categorical data between groups. Odds ratios (OR) and 95% confidence intervals (95% CI) were calculated using the binary logistic regression model. The haplotype frequencies for pairs of alleles were determined using the SHEsis program (Shi and He 2005). Linkage disequilibrium coefficients (*r* ^2^ values) for pairs of the most common alleles at each locus were estimated using SHEsis (Shi and He 2005). In case of the multiple comparisons (the genotype and haplotype analysis), Bonferroni adjustments were applied.

The results of both T cells and B cells *BTLA* mRNA expression in the CLL for different genetic variants were compared using the Mann–Whitney *U* test. The results are presented as the median and interquartile range.

## Results

### BTLA Polymorphisms Distributions in Polish Population

As we published previously (Partyka et al. 2015), two polymorphisms—rs76844316, described in the literature as polymorphic in Japanese population (Oki et al. 2011), and rs16859633, chosen on the basis of HapMap analysis—seem not to be polymorphic in Poles since none of 200 genotyped volunteers and 100 CLL pts. were carriers of mutant alleles. The distribution for the other polymorphisms is presented in Table 2.

**Table 2:** Genotypes’ distributions of *BTLA* gene polymorphisms among CLL patients and the controls

SNP	Genotype	Cases		Controls		OR	95% CI		Cases vs. controls
		*N*	%	*N*	%				
rs2705511	AA	142	44.2	263	56.0	1	–	–	*χ*_*df*=1_^2^ = 11.83 *p* = 0.0006
	AC	159	49.5	175	37.2	1.68	1.25	2.26	
	CC	20	6.2	32	6.8	1.17	0.65	2.10	
rs1982809	AA	156	48.6	279	59.4	1	–	–	*χ*_*df*=1_^2^ = 8.93 *p* = 0.0028
	AG	143	44.5	163	34.7	1.57	1.16	2.11	
	GG	22	6.9	28	6.0	1.41	0.78	2.53	
rs9288952	AA	289	90.0	415	88.3	1	–	–	*χ*_*df*=1_^2^ = 0.58 *p* = 0.4442
	AG	31	9.7	53	11.3	0.85	0.53	1.35	
	GG	1	0.3	2	0.4	0.86	0.11	6.56	
rs9288953	CC	101	31.5	185	39.4	1	–	–	*χ*_*df*=1_^2^ = 9.69 *p* = 0.0018
	CT	150	46.7	220	46.8	1.25	0.91	1.72	
	TT	70	21.8	65	13.8	1.97	1.30	2.98	
rs2705535	CC	315	98.1	456	97.0	1	–	–	*χ*_*df*=1_^2^ = 0.95 *p* = 0.3290
	CT	6	1.9	14	3.0	0.65	0.25	1.65	
	TT	0	0	0	0	1.45	0.03	73.12	
rs1844089	GG	270	84.1	389	82.8	1	–	–	*χ*_*df*=1_^2^ = 0.27 *p* = 0.6015
	GA	50	15.6	79	16.8	0.91	0.62	1.34	
	AA	1	0.3	2	0.4	0.86	0.11	6.58	
rs2705565	CC	292	91.0	421	89.6	1	–	–	*χ*_*df*=1_^2^ = 0.53 *p* = 0.4662
	CT	29	9.0	48	10.2	0.88	0.54	1.42	
	TT	0	0.0	1	0.2	0.48	0.02	11.83	
rs2633580	CC	274	85.4	393	83.6	1	–	–	*χ*_*df*=1_^2^ = 0.56 *p* = 0.4544
	CG	46	14.3	74	15.7	0.89	0.60	1.33	
	GG	1	0.3	3	0.6	0.61	0.09	4.19	
$${\chi_{*}^{2} \ge \chi^{2}_{\text{test}} = 33.3}, P= 0.00445$$									

* Distribution estimated numerically

### Hardy–Weinberg Equilibrium

No polymorphism data from the control group demonstrated any deviation from the HWE. While in the CLL group, we observed deviation from HWE for the rs2705511 with overrepresentation of heterozygotes [AC] (*f* = −0.16, *p* = 0.01). For other SNPs, there was no deviation from HWE in CLL pts. (Supplementary material 3).

### BTLA Polymorphisms and the Risk of CLL

The global distribution of investigated *BTLA* gene polymorphisms differed significantly between CLL and control groups (*p* = 0.0045) (Table 2). In particular, the differences between CLL pts. and the controls in the frequency of genotypes were observed for three polymorphic sites: rs1982809, rs2705511 and rs9288953.

For rs1982809A>G, we observed higher frequency of [AG] and [GG] genotypes among CLL pts. compared to the controls (44.5 vs 34.7 and 6.9 vs 6.0%, respectively), which indicates that the presence of [G+] allele (genotype [AG] or [GG]) increased the risk of disease about 1.5-fold compared to homozygous [AA] (OR 1.51, 95% CI 1.14–2.02, *p* = 0.004).

In case of rs2705511A>C, we observed high prevalence of persons possessing [C+] allele ([AC] or [CC] genotype) among CLL pts. compared to the controls (55.7 vs. 44.0%). The presence of this allele was associated with the increased risk of disease (OR 1.6, 95% CI 1.20–2.13, *p* = 0.0012). Moreover, the presence of [T] allele in rs9288953C>T increased the risk of CLL in a dose-dependent manner (Table 2). Individuals with rs9288953[TT] genotype were two times more prone to CLL than persons with rs9288953[CC] genotype (OR 1.97, 95% CI 1.30–2.98, *p* = 0.001), while those with rs9288953[CT] genotype conferred 25% higher risk of CLL than carriers of rs9288953[CC] genotype (OR 1.25, 95% CI 0.91–1.72, *p* = 0.17). In dominant model, possessing [T+] allele increased the risk of CLL 1.4-fold (OR 1.4, 95% CI 1.05–1.91, *p* = 0.023).

The haplotype analysis showed the presence of four haplotypes with frequency higher than 3% in healthy individuals, while in CLL group, five haplotypes have been observed at the same frequency limit (Table 3). The global distribution of haplotypes differed significantly between CLL pts. and the controls (*p*_corrected_ = 0.02). The haplotype rs2705511C/rs1982809G/rs9288952A/rs9288953T/rs2705535C/rs1844089G/rs2705565C/rs2633580C was significantly more frequently observed in CLL pts. than in the controls (18.3 vs. 12.5%, OR 1.59, 95% CI 1.20–2.11, *p*_corrected_ = 0.005). Of note, this haplotype included all alleles which were found to be associated with the risk of CLL. The haplotype rs2705511A/rs1982809A/rs9288952A/rs9288953C/rs2705535C/rs1844089G/rs2705565C/rs2633580C decreased the risk of CLL (OR 0.70, 95% CI 0.57–0.87, *p*_corrected_ = 0.006).

**Table 3:** Haplotype frequencies of the investigated *BTLA* gene polymorphisms among CLL patients and the controls (sorted by frequency in the controls)

rs2705511	rs1982809	rs9288952	rs9288953	rs2705535	rs1844089	rs2705565	rs2633580	Cases (%)	Controls (%)	OR	95% CI	*p*
**A**	**A**	A	**C**	C	G	C	C	39.6	47.3	0.70	0.56–0.87	**0.001**
A	A	A	T	C	G	C	C	22.3	20.7	1.10	0.85–1.41	0.47
**C**	**G**	A	**T**	C	G	C	C	18.3	12.5	1.58	1.20–2.10	**0.001**
C	A	A	C	C	G	C	C	3.5	3.8	0.94	0.55–1.67	0.81
C	G	A	C	C	G	C	C	3.4	2.8	1.22	0.69–2.18	0.49

*χ*_*df*=4_^2^ = is 14.45 (frequency <0.03 in both control and case has been dropped), *p* = 0.0060, *p*_corrected_ = 0.02

Statistically significant results were given in bold

### BTLA Polymorphisms and CLL Outcome

The classical prognostic parameters, such as elevation of beta 2 microglobulin (β2M) and lactate dehydrogenase (LDH), zeta-chain-associated protein kinase 70 (ZAP70), CD38, male sex and age together with *BTLA* genes polymorphisms, were analyzed in multivariate analysis in relation to TFS and OS. We have found the association between the following variables with TFS: gender (men), increased level of β2M, LDH, ZAP70 (Table 4), while men gender and β2M associated with OS. It means, for example, that increase of 10% of β2M level increases the risk of implementation of treatment of 7% (HR 1.07). None of the investigated SNPs of *BTLA* gene were associated with TFS and OS.

**Table 4:** Treatment-free survival time and overall survival time according to the clinical and genetic variables

Variable	Treatment-free survival				Overall survival			
	HR	95% CI		*p* value	HR	95% CI		*p* value
Gender—man	2.28	1.26	4.12	**0.007**	5.32	1.51	18.80	**0.009**
β2M^a^	1.07	1.01	1.13	**0.01**	1.14	1.04	1.25	**0.005**
LDH^a^	1.07	0.99	1.15	0.06	1.08	0.98	1.19	0.12
ZAP70^a^	0.98	0.96	0.99	**0.005**	1.01	0.98	1.05	0.57
CD38^a^	0.99	0.97	1.01	0.23	0.99	0.96	1.02	0.60
Age^b^	0.99	0.96	1.02	0.53	0.98	0.93	1.03	0.36
17p deletion	0.91	0.34	2.44	0.85	0.54	0.07	4.36	0.56
rs1844089 GG → GA|AA	0.93	0.61	1.43	0.757	1.80	0.65	4.97	0.26
rs9288952 AA → AG|GG	0.72	0.41	1.28	0.26	1.31	0.29	5.96	0.73
rs1982809 AA → AG	0.97	0.70	1.33	0.95	0.88	0.33	2.34	0.22
rs1982809 AA → GG	0.92	0.53	1.62		0.21	0.03	1.74	
rs2633580 CC → GC|GG	0.96	0.62	1.49	0.85	1.82	0.66	5.03	0.25
rs2705511 AA → AC	0.94	0.69	1.29	0.70	0.79	0.29	2.12	0.51
rs2705511 AA → CC	1.22	0.67	2.20		0.36	0.05	2.80	
rs2705565 CC → CT|TT	0.67	0.37	1.20	0.18	0.68	0.09	5.31	0.71
rs9288953 CC → CT	1.14	0.80	1.63	0.75	0.47	0.17	1.33	0.36
rs9288953 CC → TT	1.12	0.72	1.74		0.53	0.14	2.06	

Statistically significant results were given in bold

*HR* hazard risk

^a^Increases at 10% of value

^b^Increases at one year of age

### BTLA Polymorphisms in Relation to the mRNA Expression Level in CLL Patients

Due to technical problems, we were able to isolate appropriate amount of RNA from T cells only for 33 individuals, while in all 37 cases the isolation from B cells was successful.

**Fig. 1:**
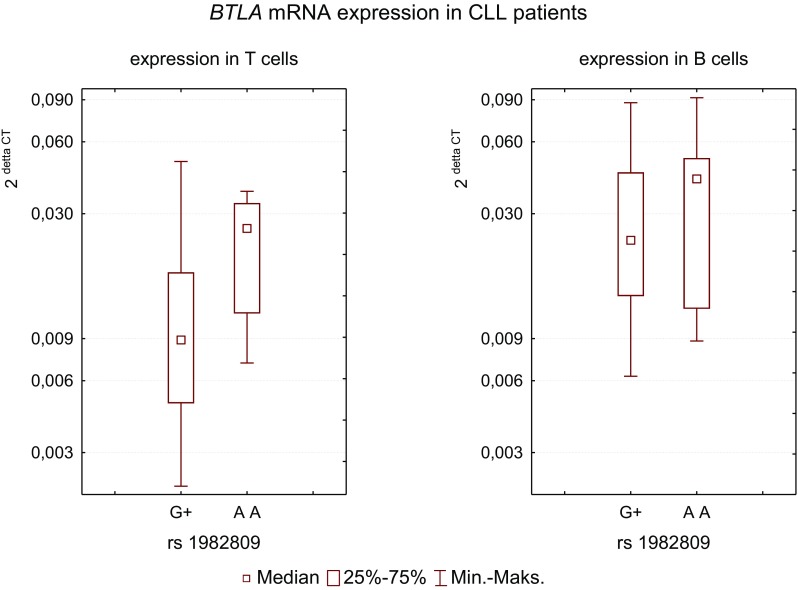
*BTLA* mRNA expression level in T and B cells in patients with CLL according to polymorphism rs1982809. Due to a low number of homozygotes GG (only two cases), the analysis was performed in two groups of patients: patients possessing [G] allele (genotype [AG] + [GG] = G+) (*n* = 24) vs. patients homozygotes [AA] (*n* = 9). The differences between median level in G+ vs. [AA] are statistically significant (0.009 ± 0.013 vs. 0.026 ± 0.012, *p* = 0.03, Mann–Whitney test), while in B cells the difference was not statistically significant (0.023 ± 0.023 vs. 0.042 ± 0.028, *p* = 0.69)

In studied groups, only two patients had genotype rs1982809[GG]; therefore, we have combined individuals with [AG] and [GG] genotypes (dominant model) into one group of patients possessing [G] allele (G+ group). The analysis of associations between *BTLA* gene polymorphisms and level of mRNA expression in T and B cells in CLL pts. showed that the presence of rs1982809[G] allele ([AG] + [GG] genotype) was associated with lower median *BTLA* mRNA expression level in T cells as compared to [AA] individuals (0.009 ± 0.013 vs. 0.026 ± 0.012, *p* = 0.032) (Fig. 1), while in B cells the difference was not statistically significant (0.023 ± 0.023 vs. 0.042 ± 0.028, *p* = 0.69).

The association with rs2705511 polymorphism which is in moderate LD with rs1982809 (*r* ^2^ = 0.577) (Partyka et al. 2015) was weaker and in individuals possessing [C] allele ([CC] + [AC] genotype—C+ group) median *BTLA* mRNA expression level in T cells was (0.008 ± 0.018 vs. 0.0165 ± 0.013, *p* = 0.09) (Supplementary material 4), while in B cells there was no difference in relation to this polymorphism (0.023 ± 0.026 vs. 0.037 ± 0.019, *p* = 0.80).

Diplotype analysis including two polymorphisms, rs1982809 and 2705511, revealed that BTLA mRNA expression levels in T cell subpopulation of CLL patients possessing predisposing rs1982809G+ and 2705511C+ alleles (*n* = 20) were significantly lower than mRNA expression levels in homozygotes [AA]/[AA] (*n* = 7) for these two SNPs (0.007 ± 0.011 vs. 0.016 ± 0.01, *p* = 0.036). However, due to the limited number of patients in group [AA]/[AA] the results should be treated with caution and repeated in larger group of patients.

For other polymorphisms, there were no relations with median *BTLA* mRNA expression level in T cells. What is more, we did not observe associations with median *BTLA* mRNA expression level in B neoplastic cells for none of the investigated SNPs (data not shown).

## Discussion

Chronic lymphocytic leukemia is one of the most prevalent leukemias in Western countries. Previously, CLL was described as a disease deriving from an inherent defect in apoptosis pathways, in which slowly proliferating B lymphocytes accumulate due to this diminished cell death. Currently, in line with recent literature, it is suggested that the population of CLL cells may also contain proliferating cells originated in the bone marrow, lymph nodes or spleen. Therefore, CLL could be considered as a disease of both proliferation and accumulation (Chiorazzi 2007). In addition to the accumulation and clonal expansion of malignant B cells, several abnormalities have been demonstrated within the non-malignant T cells population.

There has been a growing appreciation of the importance of the co-stimulatory and co-inhibitory regulation pathways, especially the potential role of BTLA/HVEM pathway in CLL (M’Hidi et al. 2009; Mocellin et al. 2013). Here, we have focused our attention on the association of genetic variation of gene encoding the most recently described co-inhibitory molecule BTLA with CLL risk and outcome.

Quite opposite to the well-known co-inhibitory molecule CTLA-4 for which the genetic variations were studied in many autoimmune and cancer diseases, the polymorphisms in *BTLA* gene were poorly explored. In our study, we have found three SNPs associated with CLL risk: rs2705511, rs1982809 and rs9288953.

The results obtained for rs2705511 corresponded with clear deviation from the HWE in the CLL pts. (*f* = −0.158; *p* = 0.0059). At the same time, the frequency of genotypes in the control group was in complete HWE (*f* = 0.011; *p* = 1). This fact may confirm the association between rs2705511 and CLL risk, since, according to Lee (2003) in the presence of an association with disease, cases do not need to be in HWE and deviation from HWE of data sets of the affected individuals is sufficient to discover the relationship with disease.

On the basis of the literature data, it was difficult to predict how rs2705511 and rs1982809 SNPs may influence the BTLA function since there were no data on the potential functional role of that polymorphisms in SNPinfo and FastSNP databases (Xu et al. 2007; Yuan et al. 2006). Both SNPs are in moderate LD (*r* ^2^ = 0.577) and are situated between genes encoding CD200 and BTLA. rs1982809 is situated in 3′ nearby gene region of *BTLA* (−101,081||−73 bp), while rs2705511 is situated in intragenic region (−97,820 bp||−3334 bp). Interestingly, CD200 is also a type-1 membrane glycoprotein, which belongs to the immunoglobulin superfamily. Animal studies of the related genes in mouse and rat suggest that *CD200* gene may regulate myeloid cell activity and delivers an inhibitory signal for the macrophage lineage in diverse tissues. CD200 is also expressed by lymphoid lineage cells such as NK cells, CD4^+^ cells and CD8^+^ cells (Gorczynski 2005). CD200 has been shown to play an important role in the regulation of anti-tumor immunity, and overexpression of CD200 has been reported in a number of hematological malignancies and solid tumors as well as on cancer stem cells (Alapat et al. 2012; Kawasaki et al. 2007; Petermann et al. 2007). In particular, the high expression of this molecule was observed in CLL. What is more, Wong et al. (2010) showed that down-regulation of CD200 expression on tumor cells may improve immunogenicity of CLL and lymphoma cells and enhances the efficacy of cell-based immunotherapies.

On the basis of the present literature, it was hard to predict if the polymorphisms rs2705511 and rs1982809 influence or not the expression of *BTLA* gene. We attempted clarification whether rs2705511 and rs1982809 influence mRNA expression level of *BTLA* gene. For this purpose, we evaluated the mRNA expression level of *BTLA* gene in the subset of T cells (CD3-positive cells) and B cells (CD19-positive cells) separated from the blood samples of CLL pts. We showed that the presence of [G] allele at rs1982809 SNP was associated with lower mRNA expression level of *BTLA* gene in the subset of T cells of the CLL pts. In the rs2705511[C] allele carriers, we also observed lower mRNA expression of *BTLA* gene in T cells, but that associations did not reach statistical significance. On the basis of the current knowledge, it is hard to explain why the presence of alleles associated with lower *BTLA* mRNA expression in T cells confers susceptibility to CLL. In the literature, only M’Hidi et al. (2009) evaluated BTLA expression in CLL pts. and these authors showed higher BTLA protein expression in reactive lymph nodes of CLL pts. In our study, we analyzed the mRNA expression in T and B cells from peripheral blood samples from CLL pts.

Interestingly, the *BTLA/CD200* deletions have recently been reported in adult B cell precursor acute lymphoblastic leukemia (ALL) pts. (Safavi et al. 2015) and in pediatric cases with Down syndrome (Lundin et al. 2012). Also, Kuster et al. (2011) showed the deletions of *BTLA* and *CD200* genes in *ETV6/RUNX1*-positive children with ALL. The authors postulate that deletions of the following genes *ETV6*, *VPREB1*, *CDKN2A/B*, *TBL1XR1*, *PAX5* as well as *BTLA* and *CD200* are very early and essential events in leukemia development. What is more in B cell precursor ALL in children, the presence of *CD200/BTLA* deletions was associated with poor treatment outcome in patients treated according to the EORTC-CLG 58951 protocol, with inferior event-free survival and overall survival (Ghazavi et al. 2015).

The third polymorphism found in our study to be associated with CLL risk was rs9288953. The presence of [T] allele in rs9288953 increased the risk of CLL in a dose-dependent manner. That SNP was previously investigated by two groups in Asian population. The first work was performed by Inuo et al. (2009) and this group found no associations between this SNP and the risk of type 1 diabetes mellitus and systemic lupus erythematosus. The second study was performed by Ge et al. (2015) and these authors found that rs9288953 SNP was associated with the risk of colorectal cancer in the Chinese population.

The potential functional role of this polymorphism is not clearly described. The rs9288953 SNP is situated in the first intron of *BTLA* gene. It was reported that the first intron is important for splicing process and may regulate gene expression more efficiently than other introns (Majewski and Ott 2002). Ge et al. (2015) postulate that according to the human splicing finder software, this SNP could activate six new splice sites in splicing enhancer motifs and break one in the silencer motif and in this way may enhance the splicing signal and strengthen the expression of BTLA. We found no associations between the mRNA expression level of *BTLA* gene in T and B cells of CLL pts. in relation to that polymorphism.

None of the other here-investigated polymorphisms were associated with susceptibility to CLL. To our best knowledge, only two other publications present data in an association between *BTLA* gene polymorphisms and cancer. In the first study (mentioned above), the authors (Ge et al. 2015) investigated the association between three SNPs in *BTLA* gene: rs1844089, rs2705535 and rs9288953 and the risk of colorectal cancer. Similar to our results, they found rs9288953 to be associated with cancer risk, but two other SNPs: rs1844089, rs2705535, not to be associated with the risk of disease. However, the authors found that the association between polymorphisms and colorectal cancer risk may be modified by the diet factors and in case of rs1844089 it was pork food intake.

The second study by Fu et al. (2010) investigated the association between the following SNPs: rs1844089, rs2705535, rs9288952, rs2633562 and rs2931761 and the risk of malignant breast cancer in Chinese women. The authors observed that rs1844089[CC], rs2705535[GG] and rs9288952[CC] homozygotes were associated with lower disease risk. Moreover, they observed the strong association between those SNPs and tumor size, estrogen and progesterone receptor expression as well as C-erbB and P53 status.

We have also conducted the analysis of an association between classical CLL prognostics factors like: β2M, LDH, ZAP70, CD3, sex and age together with *BTLA* gene polymorphisms and treatment-free survival and overall survival. Our analysis showed that polymorphisms investigated here did not influence clinical outcome of the disease.

In conclusion, we postulate that *BLTA* gene polymorphisms, especially rs1982809 SNP, influence the mRNA expression level of *BLTA* gene and are associated with the risk of CLL and are worth further studies in a larger group of CLL patients.

## Electronic supplementary material

Below is the link to the electronic supplementary material.

Supplementary material 1 (DOC 84 kb)

Supplementary material 2 (DOC 69 kb)

Supplementary material 3 (DOC 97 kb)

Supplementary material 4 (DOC 379 kb)
