# Supplementary material for: Intragenic Variations in BTLA Gene Influence mRNA Expression of BTLA Gene in Chronic Lymphocytic Leukemia Patients and Confer Susceptibility to Chronic Lymphocytic Leukemia
Source: Arch Immunol Ther Exp (Warsz). 2016 Dec 8;64(Suppl 1):137–45. doi: 10.1007/s00005-016-0430-x (PMC5334439; doi:10.1007/s00005-016-0430-x)
Supplement: Supplementary file 1 — Supplementary material 1 (DOC 84 kb) [file 5_2016_430_MOESM1_ESM.doc]

**Supplementary material 1**. Methods for determination the parameters describing CLL patients: the CD38 expression , ZAP-70 expression, 17p chromosome deletion and IGHV status.

The CD38 and ZAP-70 expression were determined by flow cytometric analysis, CLL cells were stained for CD38 antigen and ZAP-70 protein expression as described previously (Grzybowska-Izydorczyk et al. 2010;Hus et al. 2006). IGHV status was examined in the first cohort as described by van Dongen et al. 2003. Sequences were considered as unmutated if there was at least (C) 98% concordance between CLL DNA and the closest germline gene and mutated if there was below 98% concordance. 17p chromosome deletion was determined differently in the two cohorts. In the first cohort (Lodz and Warsaw) the status of the 17p deletion was established as described in (Lech-Maranda et al. 2013). In cohort from Lublin copy numbers were determined by qPCR with applying TaqMan Copy Number Assays (Life Technologies) Hs02299109_cn for *APRIL* and Hs05487075_cn for *TACI.* TaqMan Human RNase P Assay was used as a copy number reference (Life Technologies). All samples were run in triplicates. *APRIL* and *TACI* genes are located on chromosome 17p13.1 and 17p11.2, respectively. In our previous study (Jasek et al. 2015) we determined CNV for these two genes and here we used these data to determine del (17p).

van Dongen J, Langerak A, Brüggemann M et al. (2003) Design and standardization of PCR primers and protocols for detection of clonal immunoglobulin and T-cell receptor gene recombinations in suspect lymphoproliferations: report of the BIOMED-2 Concerted Action BMH4-CT98-3936. Leukemia 17:2257-2317

Grzybowska-Izydorczyk O, Cebula B, Robak T, Smolewski P (2010) Expression and prognostic significance of the inhibitor of apoptosis protein (IAP) family and its antagonists in chronic lymphocytic leukaemia. Eur. J. Cancer 46:800-810

Hus I, Podhorecka M, Bojarska-Junak A, Rolinski J, Schmitt M, Sieklucka M, Wasik-Szczepanek E, Dmoszynska A (2006) The clinical significance of ZAP-70 and CD38 expression in B-cell chronic lymphocytic leukaemia. Ann Oncol 17:683-690

Jasek M, Wagner M, Sobczynski M, Wolowiec D, Kuliczkowski K, Woszczyk D, Kielbinski M, Kusnierczyk P, Frydecka I, Karabon L (2015) Polymorphisms in genes of the BAFF/APRIL system may constitute risk factors of B-. Tissue Antigens 86:279-284

Lech-Maranda E, Mlynarski W, Grzybowska-Izydorczyk O, Borowiec M, Pastorczak A, Cebula-Obrzut B, Klimkiewicz-Wojciechowska G, Wcislo M, Majewski M, Kotkowska A, Robak T, Warzocha K (2013) Polymorphisms of TNF and IL-10 genes and clinical outcome of patients with chronic lymphocytic leukemia. Genes Chromosomes. Cancer 52:287-296
