# Supplementary material for: Intragenic Variations in BTLA Gene Influence mRNA Expression of BTLA Gene in Chronic Lymphocytic Leukemia Patients and Confer Susceptibility to Chronic Lymphocytic Leukemia
Source: Arch Immunol Ther Exp (Warsz). 2016 Dec 8;64(Suppl 1):137–45. doi: 10.1007/s00005-016-0430-x (PMC5334439; doi:10.1007/s00005-016-0430-x)
Supplement: Supplementary file 2 — Supplementary material 2 (DOC 69 kb) [file 5_2016_430_MOESM2_ESM.doc]

**Supplementary material 2**. mRNA study

The subpopulations of T and B cells were separated from frozen PBMCs from 37 CLL patients. The PBMCs were thawed and resuspended in PBS with 2% of FBS and then as the first step the positive selection of T cells (CD3 positive cells) were done according to the manufacture instruction with use of Human CD3 Selection Cocktail (cat. number #18051, StemCell Technologies), then in second step the negative selection of B neoplastic cells in remained material with use of Human B cells enrichment Kit without CD43 depletion (cat. number#19154, StemCell Technologies) was performed. Cells were counted and 1 million of cells was used for total RNA extraction according to Chomczynski’s method (Chomczynski and Mackey 1995). The concentration of total RNA was measured and then 500 ng of total RNA was reverse transcribed with the iScript cDNA Synthesis Kit (Bio‑Rad). The mRNA levels of human *BTLA* and *β2microglobulin* (*β2M*) were determined using Applied Biosystems assays (Hs00699198_m1 and Pre-developed TaqMan Assay Reagents Human β2M). The efficiency for *BTLA* mRNA and *β2M* expression were 80.3 and 81.4, respectively. All of the samples were assayed in duplicate on the 7300 Real Time PCR System (Applied Biosystems). The results were calculated according to the Δ*C_t_* method (Livak and Schmittgen 2001) applying the *β2M* gene as a reference.

Chomczynski P, Mackey K (1995) Substitution of chloroform by bromo-chloropropane in the single-step method of RNA isolation. Anal. Biochem. 225:163-164

Livak KJ, Schmittgen TD (2001) Analysis of relative gene expression data using real-time quantitative PCR and the 2(-Delta Delta C(T)) Method. Methods 25:402-408
