# Supplementary material for: Intragenic Variations in BTLA Gene Influence mRNA Expression of BTLA Gene in Chronic Lymphocytic Leukemia Patients and Confer Susceptibility to Chronic Lymphocytic Leukemia
Source: Arch Immunol Ther Exp (Warsz). 2016 Dec 8;64(Suppl 1):137–45. doi: 10.1007/s00005-016-0430-x (PMC5334439; doi:10.1007/s00005-016-0430-x)
Supplement: Supplementary file 3 — Supplementary material 3 (DOC 97 kb) [file 5_2016_430_MOESM3_ESM.doc]

**Supplementary material 3**. Hardy-Weinberg equilibrium (HWE) for *BTLA* gene polymorphisms in CLL patients and in the controls.

| **SNP** | **HWE Cases** | | **HWE Controls** | |
| --- | --- | --- | --- | --- |
|  | p | *f* | p | *f* |
| rs2705511 | 0.01 | -0.16 | 0.72 | 0.02 |
| rs1982809 | 0.18 | -0.08 | 0.52 | 0.03 |
| rs9288952 | 0.58 | 0.01 | 0.68 | 0.01 |
| rs9288953 | 0.31 | 0.06 | 1.00 | 0.00 |
| rs2705535 | 1.00 | -0.01 | 1.00 | -0.02 |
| rs1844089 | 0.71 | -0.05 | 0.56 | -0.04 |
| rs2705565 | 0.63 | -0.05 | 1.00 | -0.01 |
| rs2633580 | 0.72 | -0.04 | 1.00 | -0.01 |
