# Supplementary material for: Intragenic Variations in BTLA Gene Influence mRNA Expression of BTLA Gene in Chronic Lymphocytic Leukemia Patients and Confer Susceptibility to Chronic Lymphocytic Leukemia
Source: Arch Immunol Ther Exp (Warsz). 2016 Dec 8;64(Suppl 1):137–45. doi: 10.1007/s00005-016-0430-x (PMC5334439; doi:10.1007/s00005-016-0430-x)
Supplement: Supplementary file 4 — Supplementary material 4 (DOC 379 kb) [file 5_2016_430_MOESM4_ESM.doc]

Supplementary material 4.

**Supplementary material 4.** *BTLA* mRNA expression level in T and B cells in patients with CLL according to polymorphism rs2705511. Due to low number of homozygotes [CC] (only three cases) the analysis was performed in two groups of patients: patients possessing C+ allele (genotype [AC] + [CC] = C+) (*n*=24) vs. patients homozygotes [AA] (*n*=9). The differences between median level in C+ vs. [AA] are tended to be different (0.008+0.018 vs. 0.0165+0.013, *p*=0.09, Mann-Whitney test), while in B-cells there were no difference in relation to this polymorphism (0.023+0.026 vs. 0.037+0.019, *p*=0.80).
